# Glucagon-like peptide-1 receptor blockade impairs islet secretion and glucose metabolism in humans

**DOI:** 10.1172/JCI173495

**Published:** 2023-11-15

**Authors:** Andrew A. Welch, Rahele A. Farahani, Aoife M. Egan, Marcello C. Laurenti, Maya Zeini, Max Vella, Kent R. Bailey, Claudio Cobelli, Chiara Dalla Man, Aleksey Matveyenko, Adrian Vella

**Affiliations:** 1 Division of Endocrinology, Diabetes & Metabolism, Mayo Clinic College of Medicine, Rochester, Minnesota, USA.; 2 Division of Biomedical Statistics and Informatics, Mayo Clinic, Rochester, Minnesota, USA.; 3 Department of Women and Children’s Health and; 4 Department of Information Engineering, University of Padova, Padova, Italy.; 5 Department of Physiology and Biomedical Engineering, Mayo Clinic, Rochester, Minnesota, USA.

**Keywords:** Endocrinology, Metabolism, Beta cells, Diabetes, Insulin

## Abstract

**BACKGROUND:**

Proglucagon can be processed to glucagon-like peptide1 (GLP-1) within the islet, but its contribution to islet function in humans remains unknown. We sought to understand whether pancreatic GLP-1 alters islet function in humans and whether this is affected by type 2 diabetes.

**METHODS:**

We therefore studied individuals with and without type 2 diabetes on two occasions in random order. On one occasion, exendin 9-39, a competitive antagonist of the GLP-1 Receptor (GLP1R), was infused, while on the other, saline was infused. The tracer dilution technique ([3-^3^H] glucose) was used to measure glucose turnover during fasting and during a hyperglycemic clamp.

**RESULTS:**

Exendin 9-39 increased fasting glucose concentrations; fasting islet hormone concentrations were unchanged, but inappropriate for the higher fasting glucose observed. In people with type 2 diabetes, fasting glucagon concentrations were markedly elevated and persisted despite hyperglycemia. This impaired suppression of endogenous glucose production by hyperglycemia.

**CONCLUSION:**

These data show that GLP1R blockade impairs islet function, implying that intra-islet GLP1R activation alters islet responses to glucose and does so to a greater degree in people with type 2 diabetes.

**TRIAL REGISTRATION:**

This study was registered at ClinicalTrials.gov NCT04466618.

**FUNDING:**

The study was primarily funded by NIH NIDDK DK126206. AV is supported by DK78646, DK116231 and DK126206. CDM was supported by MIUR (Italian Minister for Education) under the initiative “Departments of Excellence” (Law 232/2016).

## Graphical abstract

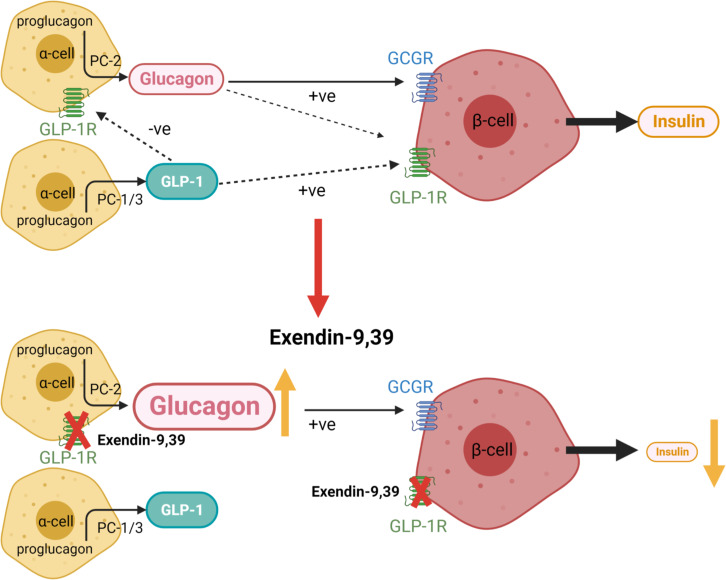

## Introduction

There are multiple strands of evidence that posttranslational processing of proglucagon in α-cells can yield glucagon-like peptide1 (GLP-1) (1). Inflammation and caloric excess (2, 3) induce prohormone convertase-1/3 (PC-1/3) in rodent (1, 3) and human islets (4). This process seems to be more established in type 2 diabetes (5, 6), leading to the suggestion that pancreatic GLP-1 acts in a paracrine fashion to support islet function.

In humans, antagonism of the GLP-1 receptor (GLP1R) during fasting with a competitive antagonist, exendin 9-39, impairs the islet cell response to an i.v. glucose challenge (7). More recently, Gray et al. (8) showed that exendin 9-39 infusion impairs the β cell response to i.v. arginine in people with and without type 2 diabetes. Inhibitors of di-peptidyl peptidase-4 (DPP-4), a ubiquitous peptidase that rapidly inactivates GLP-1, also lower fasting glucose in the absence of changes in circulating GLP-1 (8, 9). Taken together, these data show that GLP1R activation contributes to islet function in humans in the absence of increased circulating GLP-1 (as would occur after meal ingestion). This suggests that pancreatic GLP-1 supports β cell function in humans. However, it is unclear if these effects are confined to insulin secretion and are of sufficient magnitude to alter glucose metabolism, or if the effect size is altered by type 2 diabetes.

To address these questions, we studied α and β cell secretion in people with and without type 2 diabetes under fasting and hyperglycemic conditions in the presence and absence of exendin 9-39. We also used the tracer dilution technique to measure glucose turnover during the experiments. Based on exploratory experiments (Supplemental Figure 1; supplemental material available online with this article; https://doi.org/10.1172/JCI173495DS1), we hypothesized that GLP1R blockade would increase fasting glucagon and glucose concentrations in people with type 2 diabetes but not in those without diabetes. In addition, to examine whether intra-islet GLP-1 sustains islet function during metabolic stress, we restudied a subset of subjects without diabetes during acute insulin resistance induced by free fatty acid (FFA) elevation. We again used exendin 9-39 to test whether GLP1R blockade will exacerbate the deleterious effect of FFA elevation on islet function.

The other consideration is that glucagon acts as an insulin secretagogue, partially signalling through the GLP1R (10). This is another potential mechanism by which the α cell can support the β cell. Indeed, it has been suggested that glucagon elevation occurs in prediabetes and type 2 diabetes to stimulate insulin secretion by the β cell (11, 12). Whether glucagon-induced insulin secretion mediated via the GLP1R occurs to a greater extent in type 2 diabetes is unknown. Our experiment enabled us to test this hypothesis by measuring insulin secretion in response to glucagon bolus in the presence or absence of exendin 9-39.

We report that GLP1R blockade raises fasting glucose and impairs the islet response to hyperglycemia. These effects are more marked in people with type 2 diabetes where glucagon secretion, which is estimated using recently developed methodology (13), is inappropriate for the degree of hyperglycemia and impairs suppression of endogenous glucose production.

## Results

### Participant characteristics by fasting glucose and by glucose tolerance status.

A total of 12 people without diabetes and 11 people with type 2 diabetes were studied. The 2 groups were matched for age, sex, and weight at the time of screening (Table 1). Of the subjects without diabetes, none had impaired fasting glucose and 4 had impaired glucose tolerance.

### Glucose and total GLP-1 concentrations in subjects with and without type 2 diabetes.

**Figure 1:**
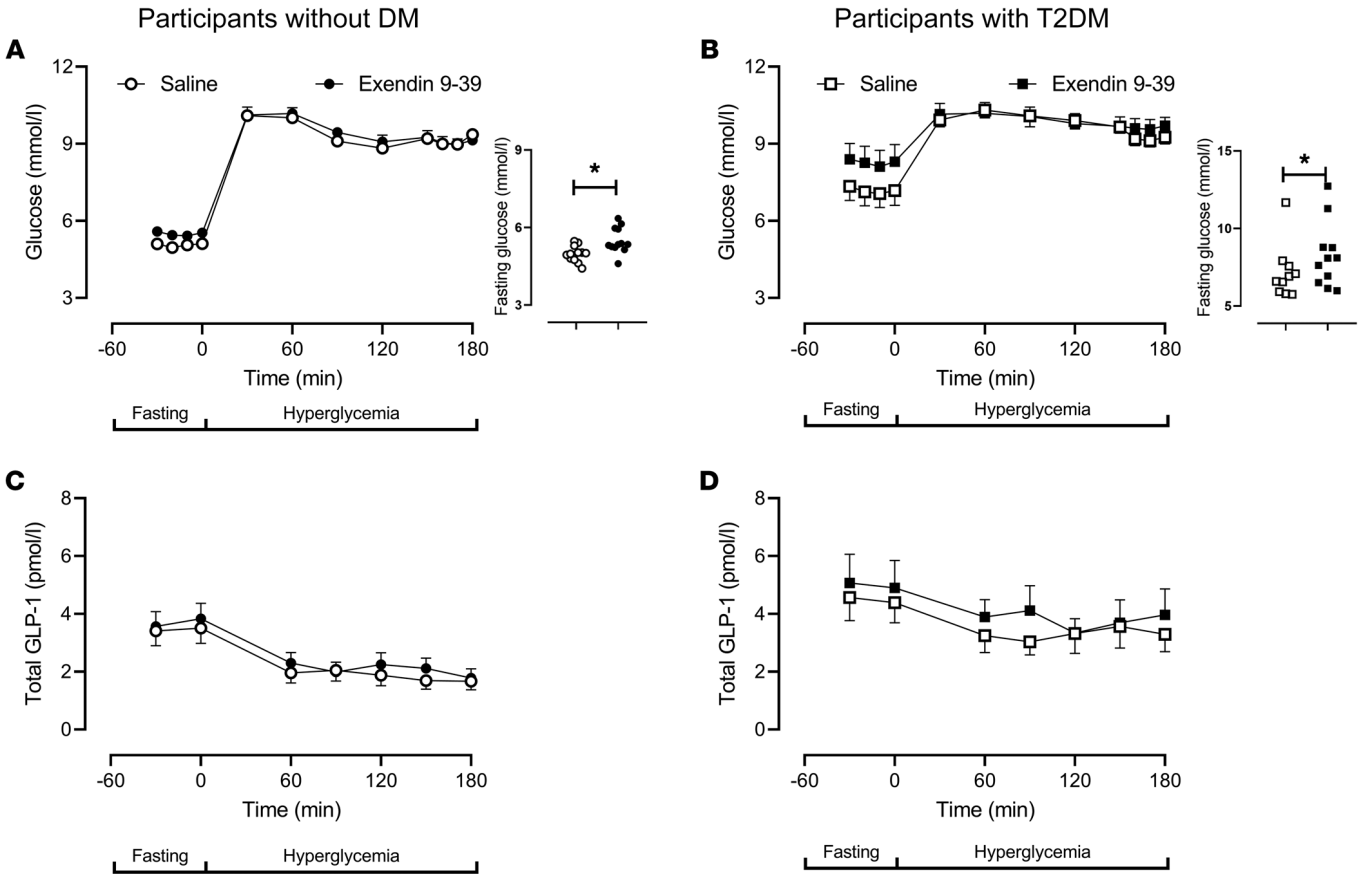
Glucose and total GLP-1 concentrations in individuals with and without type 2 diabetes. Glucose and total GLP-1 concentrations in people without diabetes (*n* = 12) during saline (open circles) and exendin 9-39 infusion (solid circles) are shown in **A** and **C**, respectively. Glucose and total GLP-1 concentrations in people with type 2 diabetes (*n* = 11) during saline (open squares) and exendin 9-39 infusion (solid squares) are shown in **B** and **D**, respectively. In **A** and **B**, smaller panels at right represent mean fasting values for each individual. Values plotted are mean ± SEM. *P <0.05 as determined by a paired Student’s *t* test.

In people without diabetes, exendin 9-39 infusion raised fasting glucose concentrations compared to saline infusion (5.0 ± 0.1 mmol/L versus. 5.5 ± 0.1 mmol/L, *P =* 0.02 [Figure 1A]). By design, glucose concentrations in the last hour of the hyperglycemic clamp did not differ between study days (8.3 ± 0.8 mmol/L versus 9.1 ± 0.2 mmol/L, *P =* 0.39). In people with type 2 diabetes, exendin 9-39 infusion also raised fasting glucose concentrations (7.2 ± 0.6 mmol/L versus 8.3 ± 0.7 mmol/L, *P* < 0.01 [Figure 1B]). By design, glucose concentrations in the last hour of the hyperglycemic clamp did not differ between study days (9.4 ± 0.2 mmol/L versus 9.7 ± 0.4 mmol/L, *P =* 0.22). Total (Figure 1, C and D) and intact GLP-1 (data not shown) were unchanged by exendin 9-39 infusion in subjects without and with type 2 diabetes, respectively.

### Insulin, C-peptide and glucagon concentrations in subjects with and without type 2 diabetes.

**Figure 2:**
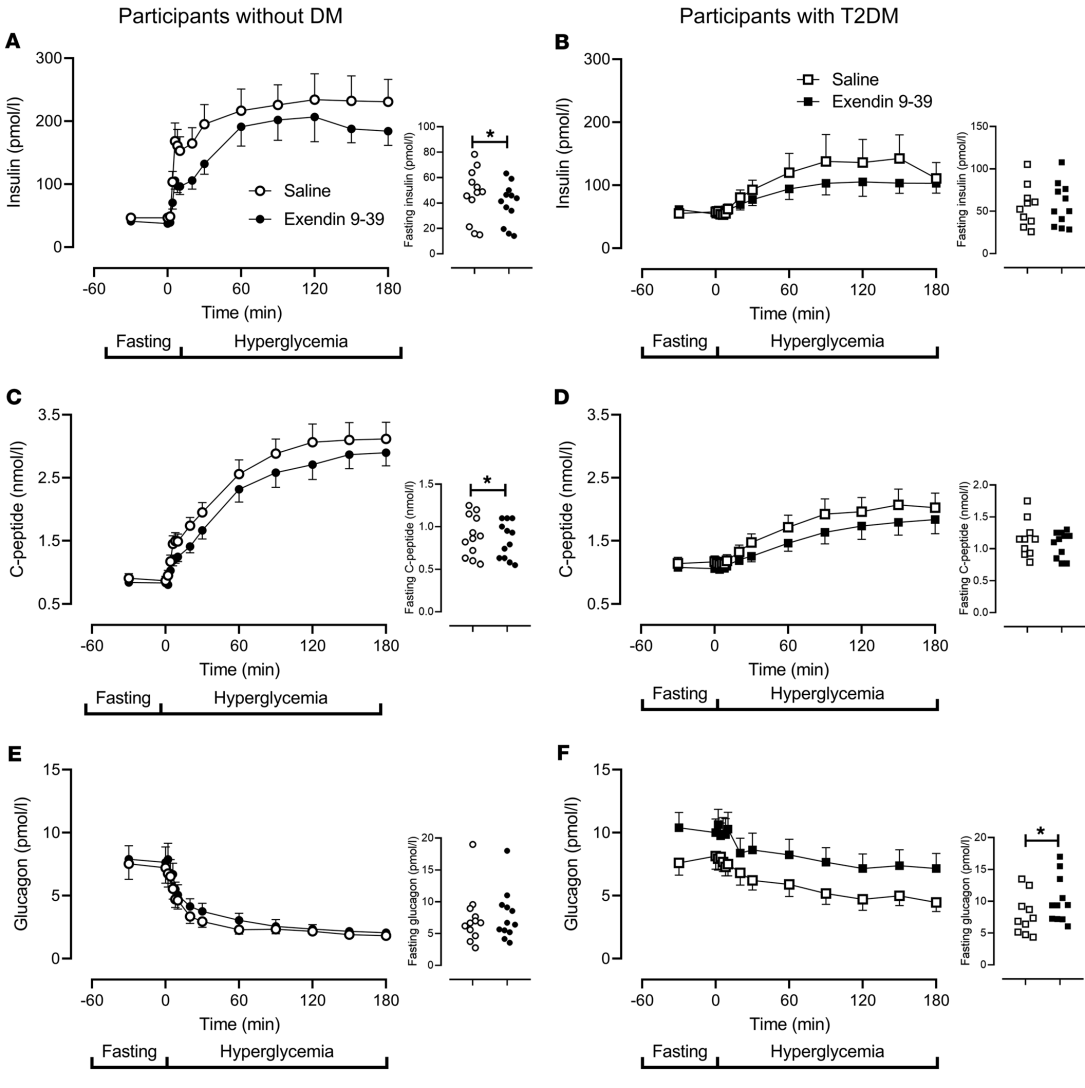
Insulin, C-peptide, and glucagon concentrations in individuals with and without type 2 diabetes. Insulin, C-peptide, and glucagon concentrations in participants without diabetes (*n* = 12) are shown in **A**, **C**, and **E**, respectively. Circles, open and filled, represent values in the presence of saline and exendin 9-39. Insulin, C-peptide, and glucagon concentrations in participants with type 2 diabetes (*n* = 11) are shown in **B**, **D**, and **F**, respectively. Squares, open and filled, represent values in the presence of saline and exendin 9-39. Values plotted are mean ± SEM. Smaller panels at right represent mean fasting values for each individual. *P <0.05 as determined by a paired Student’s *t* test.

In people without diabetes, exendin 9-39 infusion lowered fasting insulin concentrations compared to saline infusion (47 ± 6 pmol/L versus 39 ± 5 pmol/L, *P =* 0.02 [Figure 2A]). However, insulin concentrations during the hyperglycemic clamp did not differ significantly between study days. In people with type 2 diabetes, exendin 9-39 infusion did not significantly alter fasting insulin concentrations (Figure 2B), nor those observed during the hyperglycemic clamp.

In people without diabetes, exendin 9-39 infusion lowered fasting C-peptide concentrations compared with saline infusion (0.9 ± 0.1 nmol/L versus 0.8 ± 0.1 nmol/L, *P =* 0.02 [Figure 2C]) but did not significantly alter concentrations during the last hour of the clamp. In people with type 2 diabetes, exendin 9-39 infusion did not significantly alter fasting C-peptide concentrations but significantly lowered mean concentrations during the last hour of the hyperglycemic clamp (2.0 ± 0.1 nmol/L versus 1.8 ± 0.1 nmol/L, *P =* 0.01 [Figure 2D]).

**Figure 3:**
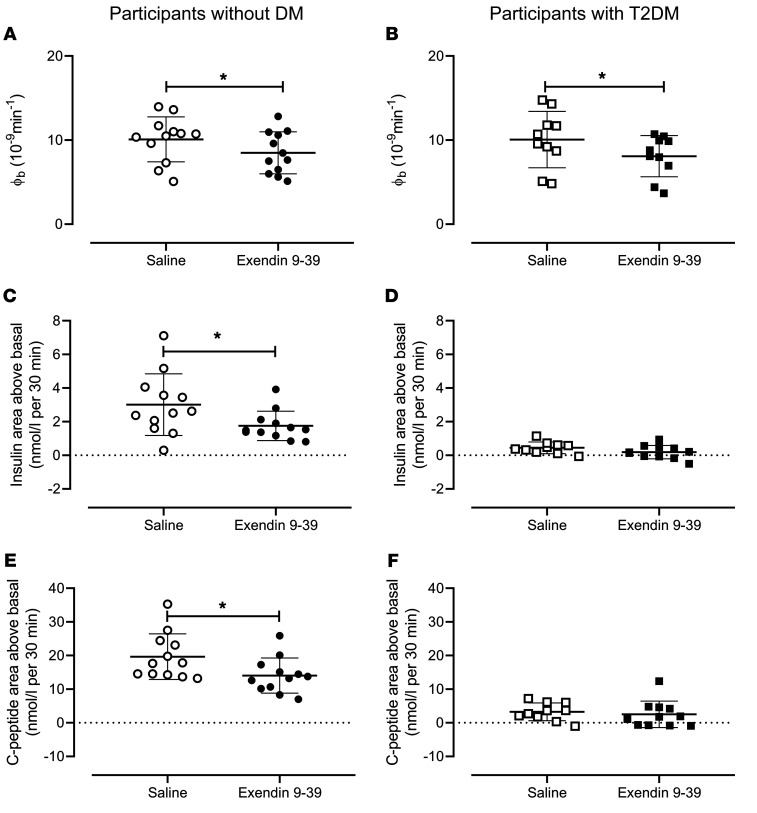
ϕ_b_, integrated initial Insulin and C-peptide responses in individuals with and without type 2 diabetes. Individual values for ϕ_b_ in individuals without diabetes (*n* = 12) are shown in **A**. AAB insulin concentrations during the first 30 minutes of the hyperglycemic clamp are shown in **C**, and AAB C-peptide concentrations are shown in **E**. Circles, open and filled, represent values in the presence of saline and exendin 9-39. The equivalent values for individuals with type 2 diabetes (*n* = 11) are shown in **B**, **D**, and **F**, respectively. Squares, open and filled, represent values in the presence of saline and exendin 9-39. Bars plotted represent mean ± SEM. *P <0.05 as determined by a paired Student’s *t* test.

Posthoc, we noted that the incremental increase in integrated insulin and C-peptide concentrations during the first 30 minutes of hyperglycemia was decreased by exendin 9-39 infusion in people without diabetes. No such effect was noted in people with type 2 diabetes (please also see Figure 3).

In people without diabetes, exendin 9-39 infusion did not change fasting or nadir glucagon concentrations during the hyperglycemic clamp (Figure 2E). In contrast, in people with type 2 diabetes, it raised fasting glucagon concentrations (7.9 ± 0.3 nmol/L versus 10.2 ± 0.3 nmol/L, *P* < 0.01 [Figure 2F]) as well as nadir concentrations (6.8 ± 0.6 nmol/L versus 4.1 ± 0.4 nmol/L, *P* < 0.01) during the hyperglycemic clamp.

### ϕ_b_, integrated initial insulin and C-peptide responses in people with and without type 2 diabetes.

Individual fasting β cell responsivity (ϕ_b_) in people without diabetes (Figure 3A) and in those with type 2 diabetes (Figure 3B) was significantly decreased by exendin 9-39 infusion. The symmetrical percent change (–18% ± 4% versus –21% ± 6 %) did not differ (*P =* 0.61) between groups.

In people without diabetes (Figure 3C), the incremental increase in integrated insulin concentrations during the first 30 minutes of hyperglycemia was decreased by exendin 9-39 infusion (3.0 ± 0.5 nmol/L versus 1.8 ± 0.3 nmol/L per 30 minutes, *P* < 0.01). This was not the case in people with type 2 diabetes (Figure 3D).

The incremental increase in integrated C-peptide concentrations during the first 30 minutes of hyperglycemia followed the same pattern (9.7 ± 2.0 nmol/L versus 14.1 ± 1.5 nmol/L per 30 minutes, *P =* 0.01 [Figure 3E]) in people without diabetes. This was not the case in people with type 2 diabetes (Figure 3F).

### Hepatic extraction, insulin secretion rates and glucagon secretion rates in people with and without type 2 diabetes.

**Figure 4:**
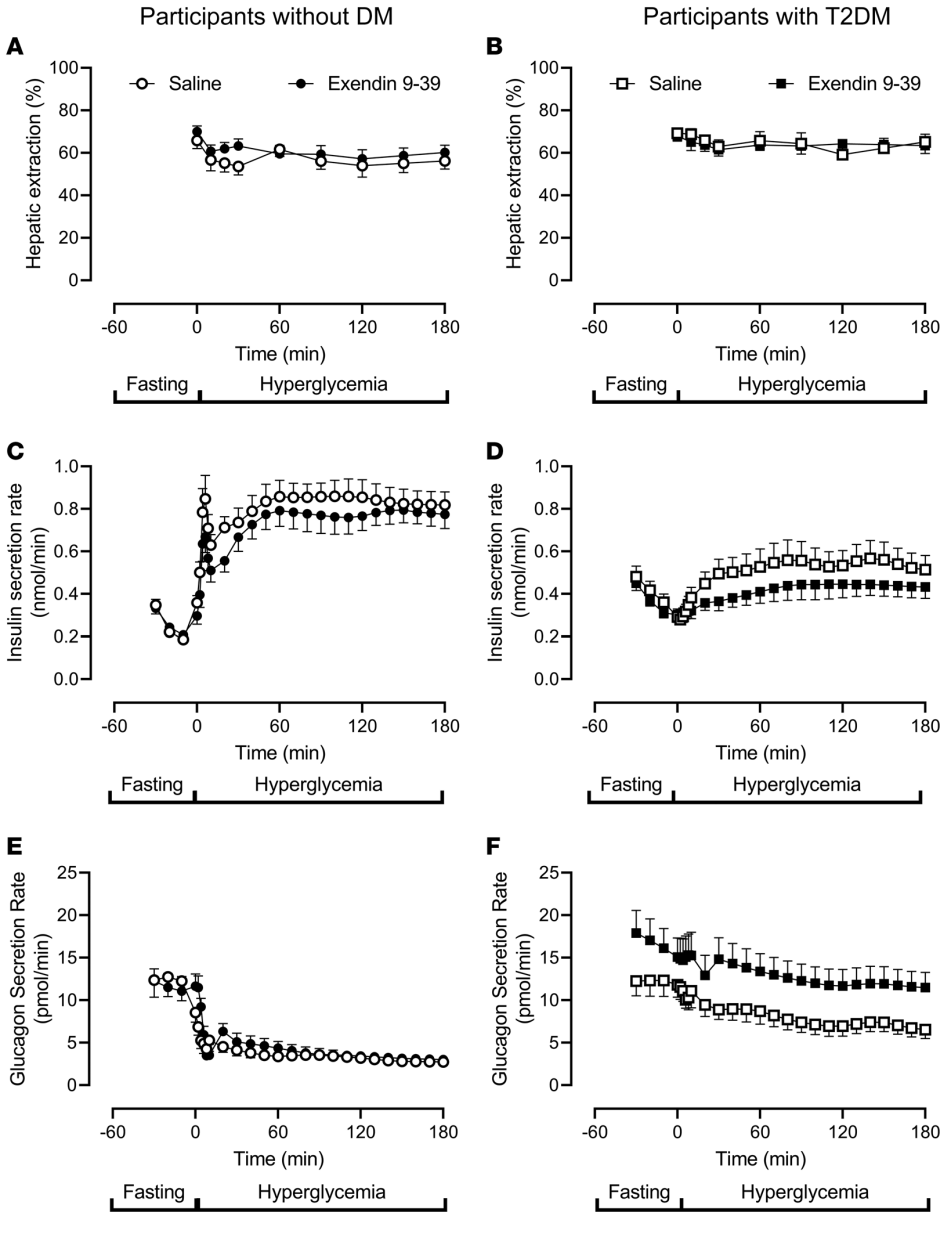
Hepatic extraction and insulin secretion rates and glucagon secretion rates in individuals with and without type 2 diabetes. The hepatic extraction of insulin, ISR,and glucagon secretion on both study days for participants without diabetes (*n* = 12) are shown in **A**, **C**, and **E**, respectively. Circles, open and filled, represent values in the presence of saline and exendin 9-39. The equivalent values for individuals with type 2 diabetes (*n* = 10) are shown in **B, D**, and **F**, respectively. Squares, open and filled, represent values in the presence of saline and exendin 9-39. Values plotted are mean ± SEM.

In people without diabetes, exendin 9-39 increased hepatic extraction of insulin slightly but significantly during fasting (0.66 ± 0.04 versus 0.70 ± 0.03, *P =* 0.04 [Figure 4A]). Hepatic extraction did not differ during the hyperglycemic clamp although posthoc testing suggested a similar decrease at 20 and 30 minutes. In people with type 2 diabetes, exendin 9-39 did not alter hepatic extraction of insulin (Figure 4B).

Fasting insulin secretion rates and insulin secretion rates during the hyperglycemic clamp were unchanged by exendin 9-39 infusion in people without diabetes (Figure 4C). On the other hand, in people with type 2 diabetes, although exendin 9-39 did not alter fasting insulin secretion, the response to hyperglycemia was decreased so that mean secretion rate in the final hour of the clamp was decreased (0.54 ± 0.07 nmol/min versus 0.44 ± 0.05 nmol/min, *P =* 0.04 [Figure 4D]) as was the integrated incremental response to hyperglycemia.

Fasting glucagon secretion rates and glucagon secretion rates during the hyperglycemic clamp were unchanged by exendin 9-39 infusion in people without diabetes (Figure 4E). On the other hand, in people with type 2 diabetes, exendin 9-39 increased fasting (12 ± 2 pmol/min versus 17 ± 2 pmol/min, *P =* 0.01) and nadir (6 ± 1 pmol/min versus 9 ± 2 pmol/min, *P =* 0.02) glucagon secretion. In addition, glucagon secretion remained significantly elevated during exendin 9-39 infusion in the final hour of the clamp (7 ± 1 pmol/min versus 12 ± 2 pmol/min, *P* < 0.01 [Figure 4F]).

### Endogenous glucose production and glucose disappearance in people with and without type 2 diabetes.

**Figure 5:**
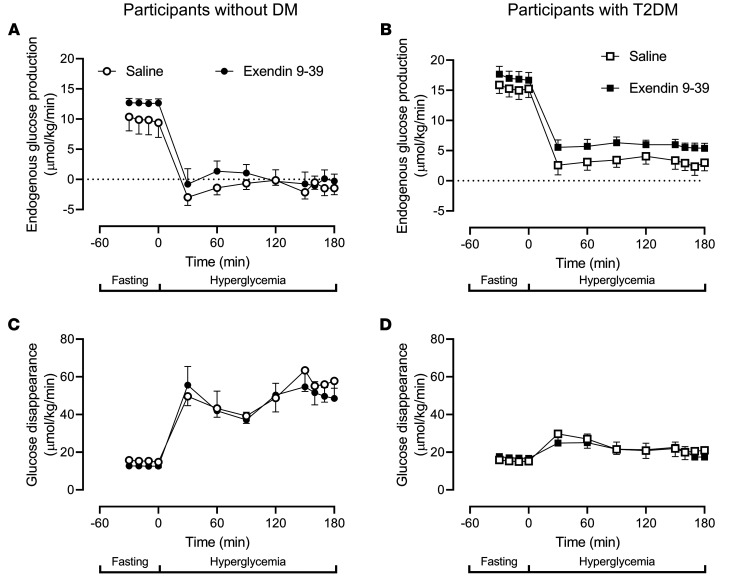
Endogenous glucose production and glucose disappearance in individuals with and without type 2 diabetes. Rates of EGP and glucose disappearance in individuals without diabetes (*n* = 12) are shown in **A** and **C**, respectively. Circles, open and filled, represent values in the presence of saline and exendin 9-39. The equivalent values for individuals with type 2 diabetes (*n* = 11) are shown in **B** and **D**. Squares, open and filled, represent values in the presence of saline and exendin 9-39. Values plotted are mean ± SEM.

In people without diabetes, exendin 9-39 infusion did not produce measurable changes in fasting endogenous glucose production (EGP; 12.2 ± 0.5 μmol/kg/min versus 12.7 ± 0.7 μmol/kg/min, *P =* 0.27). At the end of the clamp, EGP was completely suppressed by hyperglycemia and hyperinsulinemia on both study days (Figure 5A).

In people with type 2 diabetes, exendin 9-39 did not produce a significant change in fasting EGP (15.3 ± 0.4 μmol/kg/min versus 17.1 ± 0.4 μmol/kg/min, *P =* 0.21). During the clamp, EGP suppression by hyperglycemia and endogenous insulin secretion was impaired so that at the end of the clamp EGP was higher in the presence of exendin 9-39 (2.9 ± 0.4 μmol/kg/min versus 5.7 ± 0.3 μmol/kg/min, *P* < 0.01 [Figure 5B]).

In people without diabetes, exendin 9-39 infusion did not alter glucose disappearance during fasting and during the hyperglycemic clamp (Figure 5C). This pattern was also observed in people with type 2 diabetes (Figure 5D).

## Discussion

Infusion of exendin 9-39 — a competitive antagonist of the GLP1R in people without type 2 diabetes — resulted in a small but significant increase in fasting glucose concentrations. This was accompanied by a lack of reciprocal increase in fasting insulin secretion, implying impairment of fasting β cell responsivity (as borne out by a decrease in ϕ_b_). In addition, the absence of suppression of glucagon secretion in response to the rise in fasting glucose implies abnormal α cell function (14) during GLP1R blockade. In people with type 2 diabetes, fasting β cell responsivity was impaired to a similar degree as that in people without diabetes, but the increase in fasting glucose during exendin 9-39 infusion was more marked. This is likely explained by the fasting glucagon concentrations, which increased to a greater extent than was observed in people without diabetes and are inappropriate (14) for the prevailing (higher) glucose concentrations. Taken together, these data reinforce the relative importance of glucagon to the regulation of fasting glucose (14, 15). They also imply that pancreatic GLP-1 is more important to the maintenance of glucagon secretion in people with type 2 diabetes than it is in people without diabetes.

The greater dysregulation of fasting glucose and islet hormone secretion by exendin 9-39 in people with type 2 diabetes would be congruent with the observation of increased islet GLP-1 expression in islets from humans (4, 16) and animals (1, 3, 17) with diabetes. This would suggest that islet expression of GLP-1 is an adaptive response to compensate for the defects present in type 2 diabetes. The existence of this mechanism has potentially important implications. For example, genetic variation of the GLP1R that increases responsivity to exogenous GLP-1 in humans has been associated with a lower level of fasting glucose (18, 19). Further, our data would suggest that pancreatic GLP-1 plays a role in the regulation of fasting glucose concentrations by altering fasting α and β cell secretion. These findings also provide an explanation for the mechanism of action for DPP-4 inhibitors in the fasting state.

During the hyperglycemic clamp, where, by design, glucose concentrations were matched between study days, both insulin and glucagon secretion rates did not differ in people without diabetes. However, it is notable that the early insulin (and C-peptide) response to hyperglycemia — perhaps a surrogate of first-phase insulin response (20) — was impaired by exendin 9-39 infusion in this group. As shown before, in the absence of an oral challenge, there was no change in peripherally measured GLP-1 concentrations (7, 21, 22).

In contrast, in people with type 2 diabetes, abnormalities in insulin and glucagon secretion rates persisted during hyperglycemia — in the latter case, to the point that hyperglycemia and hyperinsulinemia are insufficient to suppress EGP. As expected, given the absence of a first-phase response in people with type 2 diabetes, no differences in insulin and C-peptide concentrations were observed in the initial response to glucose.

To ensure that changes in hepatic extraction of insulin do not contribute to the effects of exendin 9-39 infusion, we calculated this parameter as before (23). Exendin 9-39 slightly, but significantly, changed fasting hepatic extraction in subjects without diabetes but not in those with type 2 diabetes. These effects cannot explain the observed differences in C-peptide, or of insulin secretion rates and β cell responsivity indices derived from C-peptide concentrations. However, the mechanism by which GLP1R signaling alters hepatic extraction of insulin, and its importance, if any, will require further study.

Other investigators have reported that GLP1R signalling in the β cell enhances α cell expression of PC1/3 (24) via decreased secretion of a signaling protein (14-3-3-ζ) (25). Whether this mechanism mediates long-term benefits of treatment with GLP1R agonists or after bariatric surgery remains to be ascertained. Amino acids stimulate glucagon secretion, and, in rodents, α cell hyperplasia (26, 27). α cell dysfunction (14) and elevated concentrations of certain amino acids (28) are hallmarks or subtypes of prediabetes and diabetes, but whether amino acids stimulate PC1/3 expression and islet GLP-1 production is unknown at the present time.

We deliberately chose to use an i.v. stimulus to avoid potential confounding effects of meal-stimulated GLP-1 secretion on islet function. α cell responses to protein or fat differ from those observed in response to glucose alone (29). It is possible that, in subjects without type 2 diabetes, GLP1R blockade may produce greater abnormalities in α cell function in response to fat or protein than to glucose infusion alone. Although we were able to ascertain differences in the response to GLP1R blockade between people with and without type 2 diabetes, we may have missed defects in the α cell response to macronutrients other than glucose in people without diabetes.

Due to limitations of the experimental design, there remain some unanswered questions. For example, the participants without diabetes that we studied had a mean BMI of 32 Kg/M^2^ to match the characteristics of the participants with type 2 diabetes. We (30) and others (31) have previously suggested that impaired insulin action correlates with higher fasting glucagon secretion. On the other hand, more recent work suggests that α cell function is predominantly responsible for the set point of fasting glucose regulation (14). This abnormality observed in impaired fasting glucose was independent of weight or of insulin action. In the current experiment, all the individuals without diabetes had normal fasting glucose. Therefore, our data cannot provide insights into whether α cell function can be affected by GLP1R blockade in lean people with or without impaired fasting glucose.

The other concern is that exendin 9-39 might have inverse agonist effects on GLP1R that are independent of GLP-1, as was first demonstrated in an immortalized murine β cell line (32). Previously, we examined this possibility using an i.v. glucose challenge and exendin 9-39 infused at 30 and 300 pmol/kg/min (33). At the time we reported no effects on insulin secretion but noted a small effect on insulin action, although postchallenge glucose concentrations did not differ. No effects on α cell function were observed. These subtle effects were not observed when exendin 9-39 was used in the context of an oral challenge in people without diabetes (34). In addition, this would not explain the disparity in the effect of exendin 9-39 infusion in people with and without type 2 diabetes, at least in the fasting state, nor the other evidence we previously discussed for islet production of GLP-1.

The expression of PC1/3 necessary to process proglucagon to GLP-1 is increased in acute hyperglycemia and type 2 diabetes (1, 35). Interleukin-6 drives islet expression of PC1/3 in islets (36) and protects from the effects of a high fat diet (2). Conversely, IL-6 receptor blockade in humans decreases GLP-1 secretion (37). To explore whether acute metabolic stress caused by elevated FFA might modulate this mechanism, we studied a subset of individuals without type 2 diabetes, with and without exendin 9-39, during an infusion of intralipid and heparin. The magnitude of change in β cell function produced by exendin 9-39 was unaffected by acute FFA elevation. More importantly, this did not replicate the abnormalities of α cell function during exendin 9-39 infusion observed in people with type 2 diabetes. Whether an alternative stressor, or prolonged exposure, is necessary to induce pancreatic GLP-1 will require further study (See Supplemental Figure 2).

Glucagon is an insulin secretagogue acting on β cells in part via the GLP1R. Previously, it has been suggested that in people with type 2 diabetes, or perhaps in people with prediabetes more likely to progress to type 2 diabetes, increased glucagon secretion occurs to stimulate function in failing β cells (12). As we reported previously, in people without type 2 diabetes, exendin 9-39 — presumably through GLP1R blockade — decreases insulin secretion in response to glucagon (10). This is also now observed in people with type 2 diabetes. In this series of experiments, the β cell response to glucagon did not differ from that in people without type 2 diabetes. This suggests that, in type 2 diabetes, β cells do not have increased dependency on glucagon signaling via the GLP1R to maintain insulin secretion (Supplemental Figure 3).

We conclude from this series of experiments that, in humans, GLP1R blockade, in the absence of circulating GLP-1, impairs fasting α and β cell function, resulting in fasting hyperglycemia. In people without diabetes, these defects are no longer apparent in response to hyperglycemia, although the initial insulin secretory response to hyperglycemia is impaired. On the other hand, people with type 2 diabetes exhibit more marked abnormalities in the fasting state, and these defects persist in response to hyperglycemia. These data imply that intraislet GLP1R activation sustains islet responses to glucose, and it does so to a greater degree in people with type 2 diabetes.

## Methods

### Screening

We recruited subjects through a combination of intramural and extramural advertising. To be eligible, healthy subjects had no history of chronic illness or upper gastrointestinal surgery. Additionally, they were not taking medications that could affect glucose metabolism. Subjects with type 2 diabetes had no history of microvascular or macrovascular complications and were treated with lifestyle modification alone or in combination with metformin. Potentially eligible subjects who showed interest in participating were invited to the Clinical Research and Trials Unit (CRTU) for a screening visit. After written informed consent was obtained, participants without type 2 diabetes underwent a 2-hour 75 g oral glucose tolerance test (OGTT) to characterize their glucose tolerance status, as previously described (38). All people were instructed to follow a weight-maintenance diet containing 55% carbohydrate, 30% fat, and 15% protein for at least 3 days before the study. Body composition was measured at the time of screening using dual-energy X-ray absorptiometry (Lunar). Participant characteristics are reported in Table 1.

### Medication withdrawal

Participants with type 2 diabetes taking metformin discontinued medication for 3 weeks before, and then continued off medication for the duration of their participation in the study. While off medication, they self monitored their fasting glucose at least twice daily. Values consistently above 250 mg/dL would result in discontinuation of their participation in the study.

### Experimental design

All participants underwent 2 studies, at least 2 weeks apart in random order. Participants were admitted to the CRTU at 17:00 hours on the day before the study. After consuming a standard 10 kcal/kg caffeine-free meal, they fasted overnight. The following morning at 05:30, a dorsal hand vein was cannulated and placed in a heated Plexiglas box maintained at 55°C to allow sampling of arterialized venous blood. The contralateral forearm vein was cannulated for glucose and hormone infusions. At 06:00 (–180 minutes) a primed, (10 μCi prime, 0.1 μCi/minute continuous) infusion containing trace amounts of glucose labeled with [3-^3^H] glucose was started and continued till 09:00 (0 minutes). Subsequently, the infusion was decreased (0.03 μCi/minute) so as to mimic the anticipated pattern of fall of EGP to minimize changes in specific activity (39). At that time (09:00), another glucose infusion, also labeled with [3-^3^H] glucose, was started, and the infusion rate was varied to raise peripheral glucose concentrations to approximately 160 mg/dL.

At 07:00 (–120 minutes) exendin 9-39 was infused at 300 pmol/kg/minute and continued till the end of the study at 12:30 (210 minutes). At 12:00 (180 minutes), 1 mg glucagon was given as an i.v. bolus (40, 41). At the end of the experiment all infusions were stopped, participants consumed a meal and left the CRTU when it was safe to do so. The saline day was identical to the study described above except that between –120 and 210 minutes, 0.9% saline instead of exendin 9-39 was infused.

A subset of nondiabetic participants underwent 2 additional study days (approximately 3 months after the first 2) where at 06:00 (–180 minutes) an additional infusion of Intralipid (20%, 0.011 mL/kg/minute; Baxter Healthcare) and heparin (200 units prime, 0.2 unit/kg/minute continuous) was infused to raise circulating free fatty acids and induce insulin resistance (42).

### Analytic techniques

All blood was immediately placed on ice after collection, centrifuged at 4°C, separated, and stored at –80°C until assay. Plasma glucose concentrations were measured using a Yellow Springs glucose analyzer. Plasma insulin concentrations were measured using a chemiluminescence assay (Access Assay, Beckman). Plasma C-peptide was measured using a 2-site immunenzymatic sandwich assay (Roche Diagnostics). Glucagon was measured using a 2-site ELISA (Mercodia) in accordance with the manufacturer’s instructions. Samples for the measurement of GLP-1 were collected in protease inhibitor-containing tubes and measured using an ELISA (ALPCO Diagnostics).

### Statistics

#### Calculations.

The insulin secretion rate (ISR) was calculated from C-peptide concentrations using nonparametric deconvolution and population-based measures of C-peptide kinetics (43). Likewise, glucagon secretion rate (GSR) was calculated from the measured glucagon concentrations observed during the experiments using nonparametric deconvolution and the population model of glucagon kinetics we described recently (13). Glucose rate of disappearance (Rd) was calculated using the steady state Steele equation (44, 45). EGP was calculated as the difference between the tracer determined rate of glucose appearance and the glucose infusion rate. All infusion rates are expressed as Kg per lean body mass.

#### Statistical analysis.

All continuous data are summarized as mean ± SEM. When reporting fasting values, we utilized the mean of data obtained during fasting (–30 to 0 minutes) for each individual. Similarly, when reporting clamp values, we utilized the mean of data obtained during the final hour of the clamp (120 to 180 minutes) or in the case of tracer data, 150 to 180 minutes. Please also refer to the Supporting Data Values file provided. AUC and Area Above Basal (AAB) were calculated using the trapezoidal rule. Within-group differences attributable to study conditions were assessed using a 2-tailed Student’s paired *t* test (parametric) or Wilcoxon matched-pairs signed rank test (nonparametric). To assess between-group differences, we used a 2-tailed Student’s unpaired *t* test (parametric) or a Wilcoxon test (nonparametric). In addition, to compare changes induced by exendin 9-39 across groups in response to glucagon bolus (people without type 2 diabetes, people with type 2 diabetes, and people without type 2 diabetes + FFA elevation), we calculated the symmetric percent change (46) as 100 × Log_e_ (exendin 9-39 value / saline value). BlueSky Statistics software v. 7.10 (BlueSky Statistics LLC) and Prism 5 (GraphPad Software) were utilized for the statistical analysis. *P* < 0.05 was considered statistically significant. Our power calculation for fasting glucagon concentrations was based on observed (mean ± SD) glucagon concentration of 7.0 ± 2.4 nmol/L in people with impaired fasting glucose and impaired glucose tolerance (14). Assuming similar variability, 10 individuals with type 2 diabetes would give us the ability to detect a 1.9 pmol/L (27%) difference in fasting glucagon in response to exendin 9-39 infusion (80% power, α = 0.05).

### Data availability

All data reported in this paper is provided in an accompanying Supporting Data Values file available for download. This paper does not report original code. Any additional information required to reanalyze the data reported in this paper is available from the corresponding author upon request.

### Study approval

The Mayo Clinic IRB approved the study and associated study documents. It was subsequently registered at ClinicalTrials.gov. Exendin 9-39 was infused under an IND approved by the FDA.

## Author contributions

AAW, RAF, and AME researched data and ran the studies. MCL undertook mathematical modeling of insulin and glucagon secretion. MZ and MV assisted with data management and organization as well as with the initial data analysis. KRB supervised the statistical analyses. CC and CDM supervised the mathematical modeling, contributed to the discussion and reviewed/edited manuscript. AM conducted the in vitro work in support of the original concept and assisted with the study design, contributed to the discussion and reviewed/edited manuscript. AV designed the study, oversaw its conduct, researched data, and wrote the first draft of the manuscript. AV is the guarantor of this work and, as such, had full access to all the data in the study and takes responsibility for the integrity of the data and the accuracy of the data analysis. The order of co–first authors was determined by the time that each joined the project.

## Supplementary Material

Supplemental data

Supporting data values

## Figures and Tables

**Table 1:**
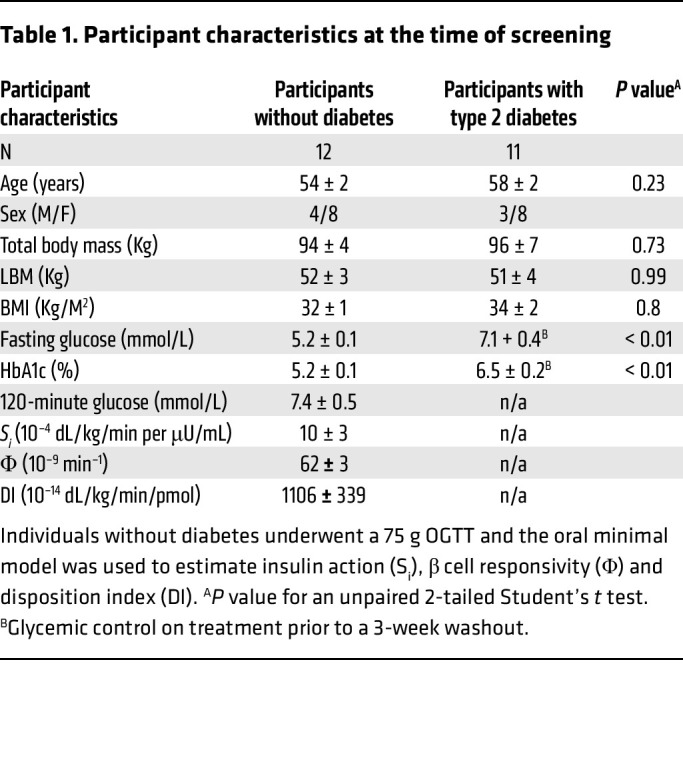
Participant characteristics at the time of screening

## Version 1 09/26/2023

In-Press Preview

## Version 2 11/15/2023

Electronic publication
